# Alpha-synuclein inclusion responsive microglia are resistant to CSF1R inhibition

**DOI:** 10.1186/s12974-024-03108-5

**Published:** 2024-04-25

**Authors:** Anna C. Stoll, Christopher J. Kemp, Joseph R. Patterson, Michael Kubik, Nathan Kuhn, Matthew Benskey, Megan F. Duffy, Kelvin C. Luk, Caryl E. Sortwell

**Affiliations:** 1 Department of Translational Neuroscience, Michigan State University, 400 Monroe Ave NW, Grand Rapids, MI 49503 USA; 2 Department of Pharmacology and Toxicology, Michigan State University, East Lansing, MI USA; 3 Center for Neurodegenerative Disease Research, Department of Pathology and Laboratory Medicine, University of Pennsylvania Perelman School of Medicine, Philadelphia, PA USA

**Keywords:** PLX3397, Neuroinflammation, Parkinson’s disease, Synucleinopathy, Major-histocompatibility complex-II, Neurodegeneration, Colony stimulating factor-1 receptor inhibition, Substantia nigra

## Abstract

**Background:**

Parkinson’s disease (PD) is a neurodegenerative disorder that is characterized by the presence of proteinaceous alpha-synuclein (α-syn) inclusions (Lewy bodies), markers of neuroinflammation and the progressive loss of nigrostriatal dopamine (DA) neurons. These pathological features can be recapitulated in vivo using the α-syn preformed fibril (PFF) model of synucleinopathy. We have previously determined that microglia proximal to PFF-induced nigral α-syn inclusions increase in soma size, upregulate major-histocompatibility complex-II (MHC-II) expression, and increase expression of a suite of inflammation-associated transcripts. This microglial response is observed months prior to degeneration, suggesting that microglia reacting to α-syn inclusion may contribute to neurodegeneration and could represent a potential target for novel therapeutics. The goal of this study was to determine whether colony stimulating factor-1 receptor (CSF1R)-mediated microglial depletion impacts the magnitude of α-syn aggregation, nigrostriatal degeneration, or the response of microglial in the context of the α-syn PFF model.

**Methods:**

Male Fischer 344 rats were injected intrastriatally with either α-syn PFFs or saline. Rats were continuously administered Pexidartinib (PLX3397B, 600 mg/kg), a CSF1R inhibitor, to deplete microglia for a period of either 2 or 6 months.

**Results:**

CSF1R inhibition resulted in significant depletion (~ 43%) of ionized calcium-binding adapter molecule 1 immunoreactive (Iba-1ir) microglia within the SNpc. However, CSF1R inhibition did not impact the increase in microglial number, soma size, number of MHC-II immunoreactive microglia or microglial expression of *Cd74*, *Cxcl10*, *Rt-1a2*, *Grn*, *Csf1r*, *Tyrobp*, and *Fcer1g* associated with phosphorylated α-syn (pSyn) nigral inclusions. Further, accumulation of pSyn and degeneration of nigral neurons was not impacted by CSF1R inhibition. Paradoxically, long term CSF1R inhibition resulted in increased soma size of remaining Iba-1ir microglia in both control and PFF rats, as well as expression of MHC-II in extranigral regions.

**Conclusions:**

Collectively, our results suggest that CSF1R inhibition does not impact the microglial response to nigral pSyn inclusions and that CSF1R inhibition is not a viable disease-modifying strategy for PD.

**Supplementary Information:**

The online version contains supplementary material available at 10.1186/s12974-024-03108-5.

## Background

Parkinson’s disease (PD), the second most common neurodegenerative disease, affects around 1 million people in the USA, with 60,000 newly diagnosed people each year [29]. Pathologically, PD is characterized by the presence of proteinaceous alpha-synuclein (α-syn) inclusions (Lewy bodies) and progressive loss of the nigrostriatal dopamine (DA) neurons [22]. While the exact cause of PD is still unknown, mounting evidence has suggested that neuroinflammation, mediated by microglia, may play a significant role in PD progression and neuropathology. Microglia have many roles in helping maintain healthy homeostasis in the brain, including synaptic pruning, neurogenesis, and neuronal surveillance [32, 39, 49]. However, microglia are main players in the immune response to an insult and allow for the bridging of the innate and adaptive immune system [5, 45]. Analysis of postmortem PD brains show increased inflammatory markers, including increases in cells immunoreactive for ionized calcium binding adaptor molecule 1 (Iba1), human leukocyte antigen (HLA-DR), and phagocytic marker CD68 in the vicinity of Lewy pathology, specifically the substantia nigra (SN) [7, 8, 19, 30, 30, 31, 31]. Patients with PD have elevated proinflammatory cytokines (i.e., interleukin 1-beta, interleukin-6, interferon gamma, and tumor necrosis factor-alpha) in their cerebrospinal fluid (CSF) and plasma, all produced by microglia and immune cells [34–37].

These pathological hallmarks of PD, namely α-syn inclusions, loss of dopaminergic neurons and neuroinflammation, can be recapitulated in vivo using the α-syn preformed fibril (PFF) model of synucleinopathy [26, 27, 41, 52]. We have previously described the time course of the accumulation of phosphorylated α-syn (pSyn) inclusions, nigrostriatal degeneration, and the microglial response in the rat PFF model [10, 41]. Specifically, the peak of pSyn inclusion formation, number of major-histocompatibility complex-II immunoreactive (MHC-IIir) microglia and microglial soma size in the substantia nigra pars compacta (SNpc) occur 2 months post intrastriatal PFF injection, months before the neurodegeneration phase occurring at 5–6 months [10, 47]. Of importance, a localized subpopulation of MHC-IIir microglia is observed immediately adjacent to nigral pSyn inclusions, with the number of responding microglia dependent on nigral inclusion load [10]. Further examination of the gene expression profile of microglia responsive to nigral pSyn inclusions has revealed upregulation of *Cd74, Cxcl10, Rt-1a2, Grn, Csf1r, Tyrobp, C3, C1qa, Serping1* and *Fcer1g*. Importantly, significant microglial upregulation of *Cd74* and *C3* was only observed following injection of α-syn PFFs, not α-syn monomer, confirming specificity of the response to α-syn aggregation [48]. These results, along with results from other laboratories [11, 17, 18, 20] suggest that pSyn inclusions are immunogenic, provoking a microglial proinflammatory response that has the potential to contribute to subsequent nigrostriatal neurodegeneration. Thus, therapeutic strategies that target and attenuate this microglial response to pathological α-syn may have potential to slow disease progression.

Pexidartinib (PLX3397B; Plexxikon inc.), a selective tyrosine kinase inhibitor, targets the macrophage (i.e. microglia) colony stimulating factor 1 receptor (CSF1R). The CSF1R is required for the activation, proliferation, and survival of microglia and, when inhibited, leads to microglial death resulting in microglial depletion within the brain parenchyma [13]. CSF1R inhibition has previously been used in mouse models of disease to understand the role microglia may play in disease progression [2, 4, 14]. However, microglia are required to maintain healthy brain homeostasis and as such, complete microglia depletion may not be a viable therapeutic strategy. Therefore, in the present study we examined the effect of CSF1R inhibitor-mediated microglia depletion on α-syn aggregation and neurodegeneration within the rat PFF model. We demonstrate that CSF1R inhibition resulted in significant, partial microglia depletion (~ 43%) of homeostatic microglia in the SNpc, but did not impact the increase in microglial number, soma size, number of MHC-II immunoreactive microglia or number of MHC-II immunoreactive microglia or expression of *Cd74, Cxcl10, Rt-1a2, Grn, Csf1r, Tyrobp,* and *Fcer1g* in microglia proximal to phosphorylated α-syn (pSyn) nigral inclusions. Further, CSF1R inhibition in the rat PFF model did not impact accumulation of pSyn and degeneration of nigral neurons. Surprisingly, long term CSF1R inhibition was associated with increased microglial soma size in remaining microglia as well as expression of MHC-II in extranigral regions. Our results do not support CSF1R inhibition as a disease modifying strategy for PD and instead suggest that long term microglial depletion may be detrimental through induction of a proinflammatory phenotype in remaining microglia.

## Methods

### Experimental overview

**Fig. 1:**
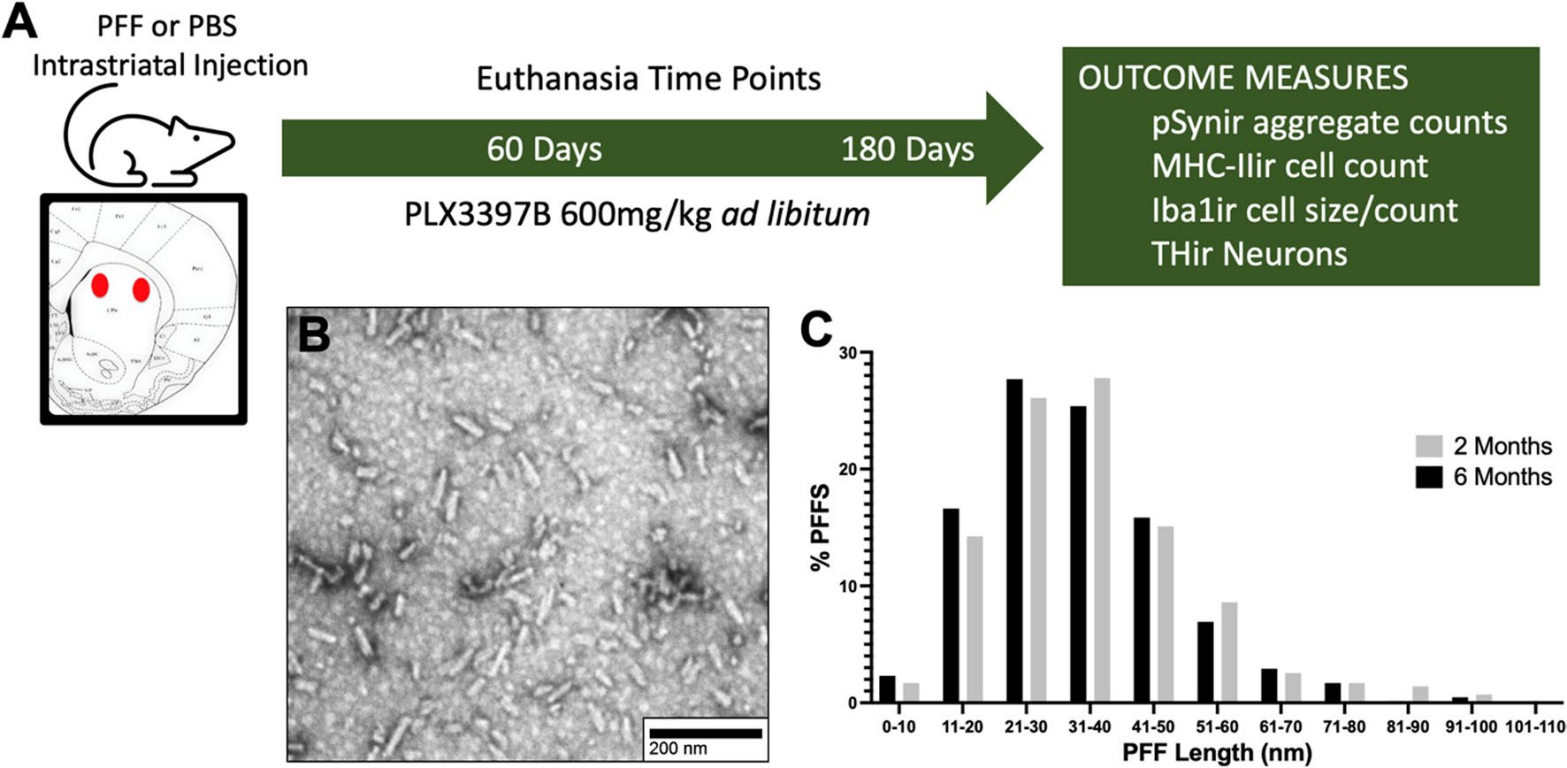
Experimental design and PFF size distribution. **A** Male Fischer 344 rats (3-months of age) received two intrastriatal injections of sonicated mouse alpha-synuclein preformed fibrils (α-syn PFFs) or phosphate buffered saline (PBS). Rats were fed Pexidartinib (PLX3397B) or control chow ad libitum starting on the day of surgery until euthanasia at 2- or 6-months post-surgery. Brains were collected for postmortem endpoints including quantification of a-syn phosphorylated at serine 129 immunoreactive (pSynir) neurons, major histocompatibility complex II immunoreactive (MHC-IIir) cells, tyrosine hydroxylase immunoreactive (THir) neurons, and ionized calcium-binding adaptor molecule 1 immunoreactive (Iba1ir) microglia count and size, in the substantia nigra pars compacta (SNpc). **B** Representative electron micrograph of sonicated α-syn fibrils. **C** Size distribution of ~ 650 sonicated fibrils prior to injection (mean fibril size-2 months: 35.9 ± 0.06 nm, 6-months: 34 ± 0.57 nm)

Rats received unilateral intrastriatal injections of either mouse α-syn PFFs or an equal volume of phosphate buffered saline (PBS) and were fed the CSF1R inhibitor PLX3397B or control chow for a period of either 60 (n = 48) or 180 days (n = 40). An additional group of rats were fed PLX3397B or control chow for 7 days prior to surgery and 60 days following surgery (n = 20). At the conclusion of the experiment rats were euthanized and brain tissue analyzed. Figure 1A illustrates the experimental design.

### Rats

Three-month old, male Fischer 344 rats (Charles River) were housed, 2–3 per cage, at the Grand Rapids Research Center vivarium which is fully approved through the Association for Assessment and Accreditation of Laboratory Animal Care (AAALAC). Rats were housed in a room with a 12-h light/dark cycle and provided food and water ad libitum*.* All procedures were done in accordance with the guidelines set by the Institutional Animal Care and Use Committee (IACUC) of Michigan State University.

### α-syn PFF preparation and fibril measurements

Α-syn PFFs were generated from wild-type-full length, recombinant mouse α-syn monomers as previously described [27, 44, 56, 57]. Quality control was completed on full length fibrils to ensure fibril formation (transmission electron microscopy), amyloid structure (thioflavin T assay), pelletability as compared to monomers (sedimentation assay), and low endotoxin contamination (*Limulus* amebocyte lysate assay; < 0.5 endotoxin units/mg of total protein). On surgery day, α-syn PFFs were thawed to room temperature and diluted to 4 µg/µl in sterile Dulbecco’s PBS and sonicated with an ultrasonic homogenizer (300 VT; Biologics, Inc.) for 60- 1 s pulses, pulser set at 20% and power output at 30%. A sample of sonicated α-syn PFFs was prepared on Formvar/carbon-coated copper grids (EMSDIASUM, FCF300-Cu). Fibrils were then imaged with a JEOL JEM-1400+ transmission electron microscope [42]. The length of ~ 650 fibrils was determined using ImageJ 1.53 K (Wayne Rasband and contributors, National Institutes of Health, USA) (Fig. 1B, C). The mean fibril length for the 2-month surgical cohort was 35.9 ± 0.06 nm and for the 6-month surgical cohort was 34 ± 0.57 nm. Fibril length < 50 nm is required for efficient seeding of endogenous α-syn inclusions [51].

### Stereotaxic injections

Unilateral intrastriatal α-syn PFF injections were conducted as previously described [41]. Rats were anesthetized with isoflurane (5% induction and 1.5% maintenance) and received unilateral intrastriatal injections to the left hemisphere (2 × 2 µl, AP + 1.6, ML + 2.0, DV − 4.0; AP + 0.1, ML + 4.2, DV − 5.0, AP and ML coordinates relative to Bregma, DV coordinates relative to dura). α-syn PFFs (4 µg/µl; 16 µg total) or an equal volume of PBS were injected at a rate of 0.5 µl/min with a pulled glass capillary tube attached to a 10 µl Hamilton syringe [42]. To avoid α-syn PFF displacement, the needle was left in place for 1 min following injection, retracted 0.5 mm and left for 2 min before fully retracted. All animals received analgesic (1.2 mg/kg of sustained release buprenorphine) after surgery and were monitored until euthanasia.

### Pexidartinib dosing

Pexidartinib chow was generously provided by Plexxikon, Inc. Rats were fed Pexidartinib binary chow (PLX3397B, 600 mg/kg; Plexxikon Inc.; Research Diets Inc.) or control chow ad libitum for either 60 or 180 days starting on the day of PFF injections. To investigate the impact of CSF1R inhibition prior to α-syn PFF injection, an additional group of rats was fed Pexidartinib (non-binary) chow or control chow ad libitum for the 7 days leading up to α-syn PFF injections and continued for 60 days until sacrifice. Rat weights and collective cage food intake was tracked weekly (Additional file 1: Figures S1A, B, Additional file 2: Figure S2A, B).

### Euthanasia

Rats were euthanized at 60 days (peak pSyn accumulation in the SNpc) or 180 days (peak nigral degeneration) post-surgery, pathological intervals that have been previously identified in this model [10, 41, 43]. Rats were given a 30 mg/kg pentobarbital injection (i.p.) (Euthanasia-III Solution, MED-PHARMEX, Inc.) and perfused intracardially with heparinized 0.9% saline. Livers were removed and weighed (Additional file 1: Figure S1C, Additional file 2: Figure S2C). Brains were removed and post-fixed in 4% paraformaldehyde (PFA) for one week and then transferred to 30% sucrose in 0.1 M phosphate buffer until sunk. Brains were frozen on dry ice and cut at 40 µm on a sliding microtome, sections were stored in cryoprotectant (30% sucrose, 30% ethylene glycol, in 0.1 M Phosphate Buffer (PB), pH 7.3) at − 20 °C.

### Immunohistochemistry

Free floating sections were washed 4 × 5 min in 0.1 M tris buffered saline (TBS) containing 0.5% Triton-X100 (TBS-Tx), quenched in 3% H_2_O_2_ for 1 h, blocked in 10% normal goat serum (NGS) in TBX-Tx, and incubated overnight in primary antibody in 1% NGS/TBS-Tx at 4 °C on a shaker. Primary antibodies used included: mouse anti-α-syn phosphorylated at serine 129 (pSyn) (1:10,000; Abcam, AB184674), mouse anti-tyrosine hydroxylase (TH) (1:4000; Millipore, MAB318), rabbit anti-ionized calcium binding adaptor molecule 1 (Iba1) (1:1000; Wako, 019-09741), mouse anti-major histocompatibility complex-II (MHC Class II RT1B clone OX-6) (1:2000; BioRad, MCA46G). Sections were washed in TBS-Tx and then incubated for 2-h at room temperature with biotinylated secondary antibodies in 1% NGS/TBS-Tx. Secondary antibodies used included: goat anti-mouse IgG (1:500; Millipore, AP124B), goat anti-rabbit IgG (1:500, Millipore, AP132B), and horse anti-mouse IgG rat preabsorbed (for TH; 1:500; Vector Laboratories, BA-2001). Sections were washed 4 × 5 min in TBS-Tx and incubated in standard avidin–biotin complex detection kit (ABC, Vector Laboratories, PK-6100). Visualization for pSyn was done using 2.5 mg/ml nickel ammonium sulfate hexahydrate (Fisher, N48-500), 0.5 mg/ml diaminobenzidine (Sigma-Aldrich, D5637), and 0.03% H_2_O_2_ in TBS-Tx. TH was visualized with 0.5 mg/ml diaminobenzidine (Sigma-Aldrich, D5637), and 0.03% H_2_O_2_ in TBS-Tx. MHC-II was visualized using Vector ImmPACT DAB (brown) Peroxidase kit (Vector Laboratories; SK-4105). Iba1 was visualized with ImmPACT VIP (purple) Peroxidase Kit (Vector Laboratories; SK-4605). Sections were mounted, allowed to dry, rehydrated, then dehydrated in ascending ethanol washes and cleared with xylene before cover slipping using Epredia Cytoseal-60 (Thermo-Fisher, 22-050-262). pSyn sections were counterstained with cresyl violet before dehydration.

### Immunofluorescence

Free floating sections were washed 5 × 5 min in TBS-Tx, blocked in 10% NGS in TBX-Tx, then incubated overnight in primary antibodies in 1% NGS/TBS-Tx at 4 °C on a shaker. Primary antibodies used included: mouse anti-pSyn (1:10,000; Abcam, AB184674) and rabbit anti-Iba1 (1:1000; Wako, 019–09741). Sections were washed in TBS-Tx and then incubated for 2-h, in the dark, at room temperature, with fluorescent conjugated secondary antibodies in 1%NGS/TBS-Tx. Secondary antibodies used included: Alexa Fluor 568 goat anti-mouse IgG (1:500, Invitrogen, A-11004), and Alexa Fluor 647 goat anti rabbit IgG (1:500, Invitrogen, A32733). Sections were then rinsed 5 × 5 min in TBS-Tx, incubated 1 × 5 min in 4ʹ,6-Diamidino-2-Phenylindole, Dihydrochloride (DAPI) made in TBS-Tx (1:10,000, Invitrogen, D1306) and placed back in TBS-Tx for mounting. Sections were mounted and cover-slipped with VECTASHIELD Vibrance antifade mounting medium (Vector Laboratories, H-1700) and kept in the dark until imaging utilizing the Zeiss Axioscan.Z1 scanning microscope.

### Total enumeration for pSyn and MHC-II

Due to heterogeneity in the distribution of both pSyn and MHC-II immunoreactive (MHC-IIir) profiles within the SN, total enumeration rather than stereological counting frames was used for quantification. The investigator was blinded to treatment groups. Total enumeration of pSyn immunoreactive (pSynir) neurons and MHC-IIir cells was conducted utilizing Microbrightfield Stereoinvestigator (MBF Bioscience). Sections containing the SN pars compacta (SNpc, 1:6 series) were used. Contours were drawn around the SNpc at 4X, a 20× magnification was then used for identification and counting. Counts represent the raw total number multiplied by six. Data are reported as total estimates of pSynir neurons or MHC-IIir cells in each hemisphere.

### Stereological assessment of nigral TH immunoreactive neurons

The number of THir neurons in the ipsilateral and contralateral SNpc was estimated using unbiased stereology with the optical fractionator principle. The investigator was blinded to treatment groups. Using a Nikon Eclipse 80i microscope, Retiga 4000R camera (QImaging) and Microbrightfield StereoInvestigator software (Microbrightfield Bioscience, Williston, VT), THir neuron quantification was completed by drawing a contour around the SNpc borders using the 4X objective on every sixth section and counting neurons according to stereological principles at 60X magnification. Briefly, counting frames (50 µm × 50 µm) were systematically and randomly distributed over a grid (183 µm × 122 µm) overlaid on the SNpc. A coefficient of error < 0.10 was accepted. Data are reported as total estimate of THir neurons in each hemisphere.

### Microglia soma size and number

Nigral sections were fluorescently labeled for pSyn and Iba1. The investigator was blinded to treatment groups. Utilizing the Zeiss Axioscan.Z1 scanning microscope, Z-Stacks images at 20X were obtained and three consecutive nigral sections representing the sections with the highest number of pSynir neurons were analyzed with Nikon Elements AR (Version 4.50.00, Melville, NY). All Iba1 immunoreactivity (Iba1ir) somas were outlined, excluding processes, and the number of individual microglial objects calculated. Data for soma size are reported as the number of pixels per outlined microglia soma. The HALO® (Indica Labs) image analysis module “Area quantification v1.0 for brightfield” was used to calculate total pSyn signal in the striatum and MHC-II signal in the mesencephalon.

### RNAscope™ HiPlex fluorescent in situ hybridization combined with immunofluorescence

RNAscope™ HiPlex Fluorescent in situ hybridization (FISH) was performed on nigral tissue sections to analyze the proinflammatory status of the remaining microglia after partial depletion. RNAscope probes were designed and produced by ACD Bio and FISH was performed as previously described [48]. Free floating sections were washed 4 × 10 min in TBS-Tx and then quenched in ACD Bio Hydrogen Peroxide (Advanced Cell Diagnostics, 322335) for 1 h. Tissue was then washed 4 × 10 min in TBS-Tx, followed by 2 × 10-min washes in TBS-Tx diluted 1:4 in ultra-pure water. Tissue was mounted on HistoBond + slides (VWR VistaVision, 16004-406) and placed on a slide warmer at 60 °C overnight. The slides were incubated in an ACD RNAscope™ Target Retrieval buffer (diluted 1:10 in ultra-pure water,Advanced Cell Diagnostics, 322001) warmed to 99 °C for 10 min and then quickly washed 2 × 1 min in ultra-pure water. Tissue sections were outlined with a Super PapPen (IHC World; SPM0928) and 3 drops of ACD protease III (Advanced Cell Diagnostics; 322337) was added and incubated in a Hybez ™II oven at 40.0 °C (Advanced Cell Diagnostics) for 30 min. Slides were then quickly washed 2 × 1 min in ultra-pure water, and diluted ACD probes (1:50; see Additional file 5: Table S1 for detailed probe information) were added to the tissue and incubated in the Hybez ™II oven for 2-h. 3 × 30-min amplification steps were done with ACD amplification buffers 1, 2, and 3 respectively [RNAscope™HiPlex12 Detection Reagents (488, 550, 650) v2; Advanced Cell Diagnostics, 324410] in a Hybez ™II oven. Between each amplification tissue was washed 2 × 1-min in RNAscope™Wash Buffer (1:500 Dilution in ultra-pure water; Advanced Cell Diagnostics, 310091). Following the 3rd amplification incubation, slides were washed 2 × 1 min and incubated for 15 min in the Hybez oven with the appropriate ACD fluorophores for the tails on the probes [RNAscope™ HiPlex12 Detection Reagents (488,550,650) v2; Advanced Cell Diagnostics, 324410]. Slides were washed 2 × 1 min and blocked in 10% NGS in TBX-Tx for 1-h at room temperature. Sections were then incubated with primary antibody (Iba1; 1:100; Wako, 019-09741) diluted in TBS-Tx containing 1% NGS overnight at RT. Slides were washed 2 × 1 min in TBS-Tx and incubated in Alexa Fluor 488-goat anti rabbit (1:250; Invitrogen, A11034) diluted in TBS-Tx containing 1% NGS for 2 h at RT. Slides were washed 2 × 1 min in TBS-Tx and a drop of RNAscope™HiPlex DAPI (Advanced cell Diagnostics; 324420) was added and left for 1 min. Excess DAPI was removed, and slides were cover slipped with ProLong™ Gold antifade reagent (Invitrogen, P36930). Images were taken using Nikon Eclipse Ni-U microscope with CFI60 infinity optical system (Nikon Instruments Inc.).

### Statistical analysis

All statistical tests were completed using GraphPad Prism software (version 9, GraphPad, La Jolla, CA). Outliers were assessed via the absolute deviation from the median method [24] utilizing the very conservative difference of 2.5X median absolute deviation as the exclusion criterion. Statistical significance was set to α ≤ 0.05. Comparisons were made across all groups using two-way analysis of variance (ANOVA) with a *post-hoc* Tukey test with the following exceptions: two-way ANOVA with repeated measures was used for comparisons of food intake over time, Student’s T-test (two-tailed) was used for comparisons in pSyn accumulation in the striatum between PFF injected PLX3397B and control rats, two-way ANOVA with *post-hoc* Tukey test comparisons in THir neurons in the SNpc were made within each brain hemisphere separately.

## Results

### Impact of CSF1R inhibition during peak aggregation in the SNpc

#### Two months of Pexidartinib (PLX3397B) partially depletes microglia in both α-syn PFF and PBS injected rats

**Fig. 2:**
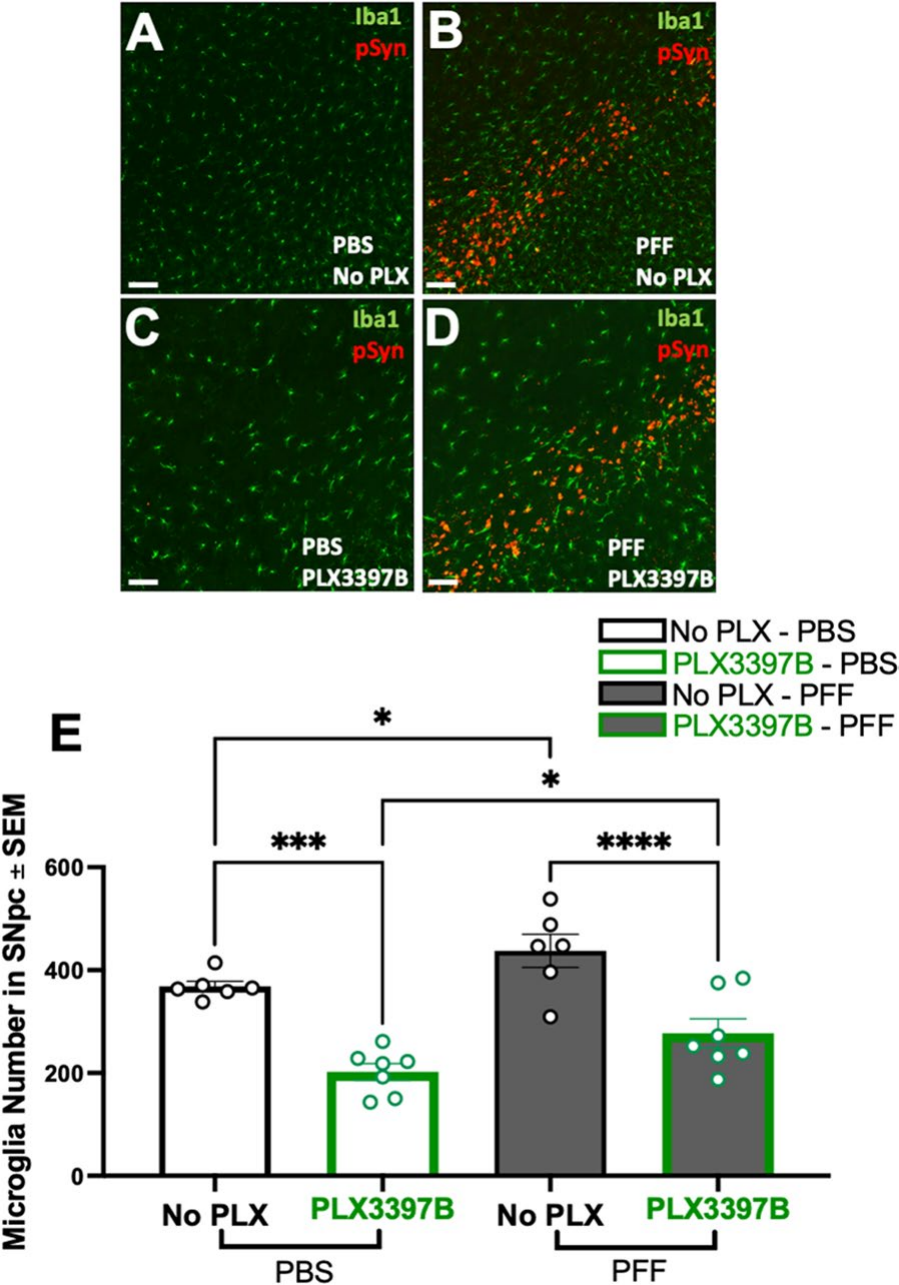
Inclusion-associated increases in microglia persist in the SNpc despite CSF1R inhibition. **A**–**D** Ionized calcium binding adaptor molecule 1 (Iba1, green) and phosphorylated alpha synuclein at serine 129 (pSyn, red) immunofluorescence in the substantia nigra pars compacta (SNpc) 2 months post intrastriatal alpha-synuclein preformed fibril (α-syn PFF) or phosphate buffered saline (PBS) injection, with or without Pexidartinib (PLX3397B) treatment. **E** Quantitation of Iba1 immunoreactive microglia in the SNpc in all treatment groups. PFF injected rats display significantly more microglia in the SNpc in both chow treatment groups. PLX3397B treatment resulted in significant microglial depletion in both PBS and PFF rats (p ≤ 0.001). Black outline = no PLX3397B; green outline = PLX3397B; *p < 0.04; ***p = 0.0001; ****p < 0.0001. Values represent the mean ± SEM. Scale bars in **A**–**D** are 100 µm

Α-syn PFF injected rats displayed substantial accumulation of pSyn within the SNpc ipsilateral to α-syn PFF injection as well as significantly more microglia compared to PBS rats, regardless of chow treatment (p < 0.04, Fig. 2A–E). Specifically, α-syn PFF injection was associated with ~ 19% and ~ 37% more microglia in the SNpc of control and PLX3397B chow rats, respectively. Treatment with Pexidartinib (PLX3397B; 600 mg/kg) for 2 months led to a significant depletion of microglia within the SNpc in both PBS and α-syn PFF injected rats. PBS PLX3397B rats displayed 45% fewer microglia (p = 0.001) and α-syn PFF PLX3397B rats displayed 36.6% fewer microglia (p < 0.001) compared to the control fed rats in their respective surgical treatment groups (Fig. 2E). These data suggest that inclusion-associated increases in microglia persist despite significant depletion of microglia due to 2 months of PLX3397B treatment.

#### CSF1R inhibition does not impact accumulation of pSyn aggregates in nigral neurons or early loss of TH phenotype

**Fig. 3:**
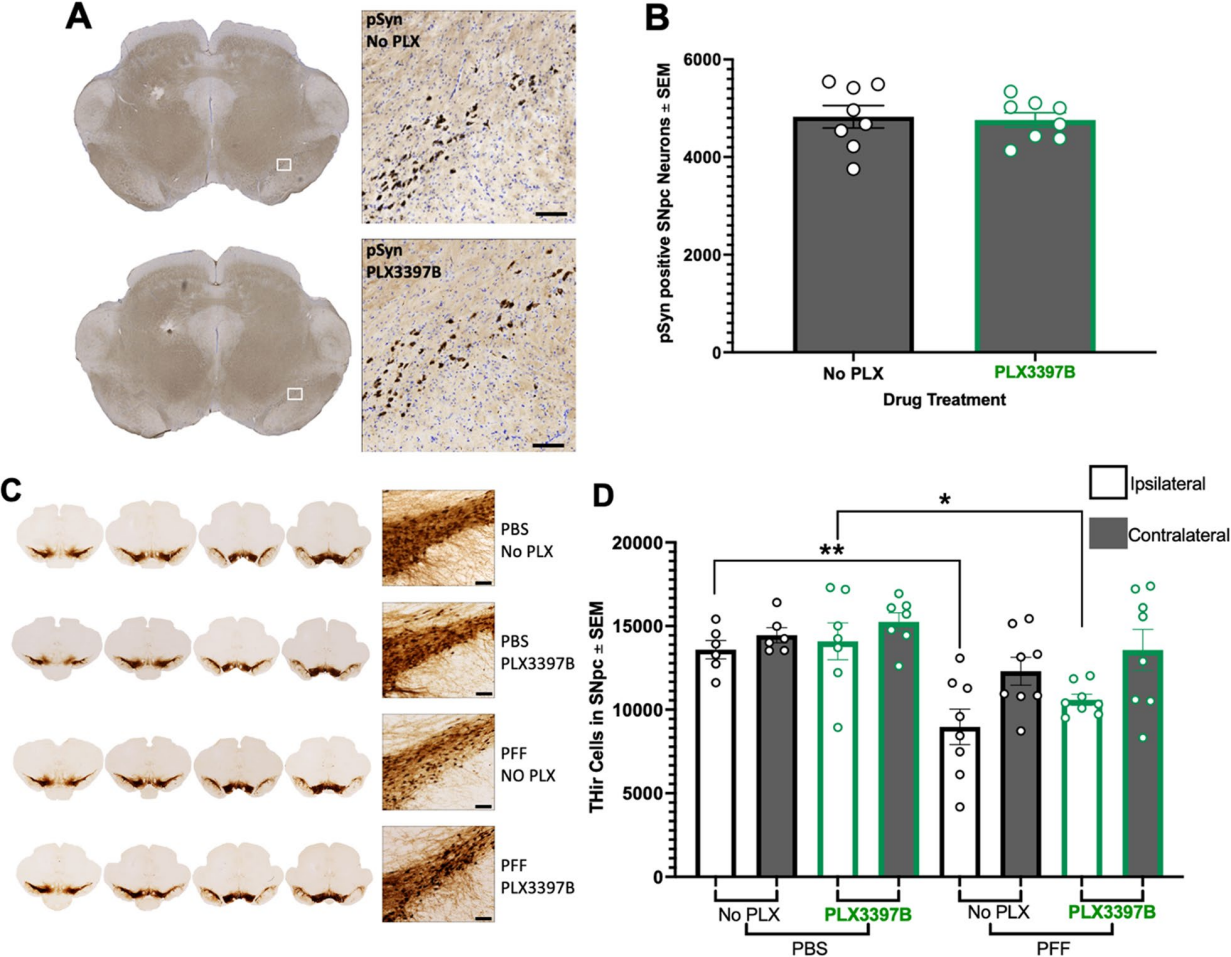
CSF1R inhibition does not impact pSyn aggregation or early loss of TH-immunoreactivity in the SNpc. **A** Phosphorylated alpha synuclein (pSyn) inclusions in the ipsilateral substantia nigra pars compacta (SNpc) 2 months post alpha synuclein preformed fibril (α-syn PFF) injection in both Pexidartinib (PLX3397B) and control fed rats. **B** Quantification of pSyn immunoreactive (pSynir) neurons in the ipsilateral SNpc 2 months after α-syn PFF injection in control and PLX3397B rats. PLX3397B treatment had no impact on the number of pSynir neurons within the SNpc. **C** Tyrosine hydroxylase immunoreactive (THir) neurons in the SNpc of α-syn PFF and control phosphate buffered saline (PBS) injected rats, with and without PLX3397B treatment. **D** Quantification of THir neurons in the SNpc 2 months following injection. PFF injected rats possessed significantly fewer THir neurons in the ipsilateral SNpc as compared to the ipsilateral SNpc of PBS injected rats. No differences in THir neurons were observed due to PLX3397B treatment. Values represent the mean ± SEM. Black outline = no PLX3397B; green outline = PLX3397B; *p = 0.0330; **p = 0.0058. Scale bars in **A** and **C** are 100 µm

Intrastriatal injection of mouse α-syn PFFs results in peak pSyn accumulation in the ipsilateral SNpc at 2 months [10, 41, 47]. In the present study we observed pSyn accumulation in the ipsilateral SNpc of α-syn PFF injected rats (Fig. 2B, D, Fig. 3A) but not in PBS control rats (Fig. 2A, C). PLX3397B treatment had no impact on the number of pSynir neurons within the SNpc of α-syn PFF rats (p > 0.05, Fig. 3B). α-syn PFF rats fed control chow possessed 4826 ± 229.3 pSyn containing neurons in the ipsilateral SNpc whereas α-syn PFF PLX3397B rats possessed 4760 ± 148.8. To investigate whether CSF1R inhibition prior to α-syn PFF injection impacted pSyn accumulation, rats were pretreated with Pexidartinib (non-binary) for one week prior to α-syn PFF injection, along with continued treatment for 2 months post injection. Pre and post treatment with Pexidartinib (non-binary) did not lead to a significant difference (p > 0.05) in the number of pSynir neurons within the SNpc (4490 ± 361.2) as compared to rats only receiving Pexidartinib (non-binary) post α-syn PFF injections (4370 ± 242.3) or control fed rats (3752 ± 442.5; Additional file 3: Figure S3A).

We next examined whether α-syn PFF injection or PLX3397B treatment for 2 months impacted THir neurons in the SNpc. Utilizing identical PFF surgical parameters in rats we have previously observed ~ 0–25% loss of THir SNpc neurons at 2 months after α-syn PFF injection, however parallel neuronal counts revealed that this represents loss of TH phenotype in the absence of overt degeneration [33, 41]. In the present study, 2 months following α-syn PFF injection we observed a 24–33% reduction (p < 0.04) in THir neurons in the ipsilateral SNpc as compared to the ipsilateral SNpc of PBS injected rats (Fig. 3C, D) both with and without PLX3397B treatment. No differences in THir neurons were observed due to PLX3397B treatment (p > 0.05). These results suggest that CSF1R inhibition does not impact THir neurons in control rats, nor does it prevent the modest loss of TH phenotype associated with the aggregation phase of the PFF model.

#### CSF1R inhibition does not impact reactive microglia morphology or MHC-II expression associated with α-syn inclusions in the SNpc

**Fig. 4:**
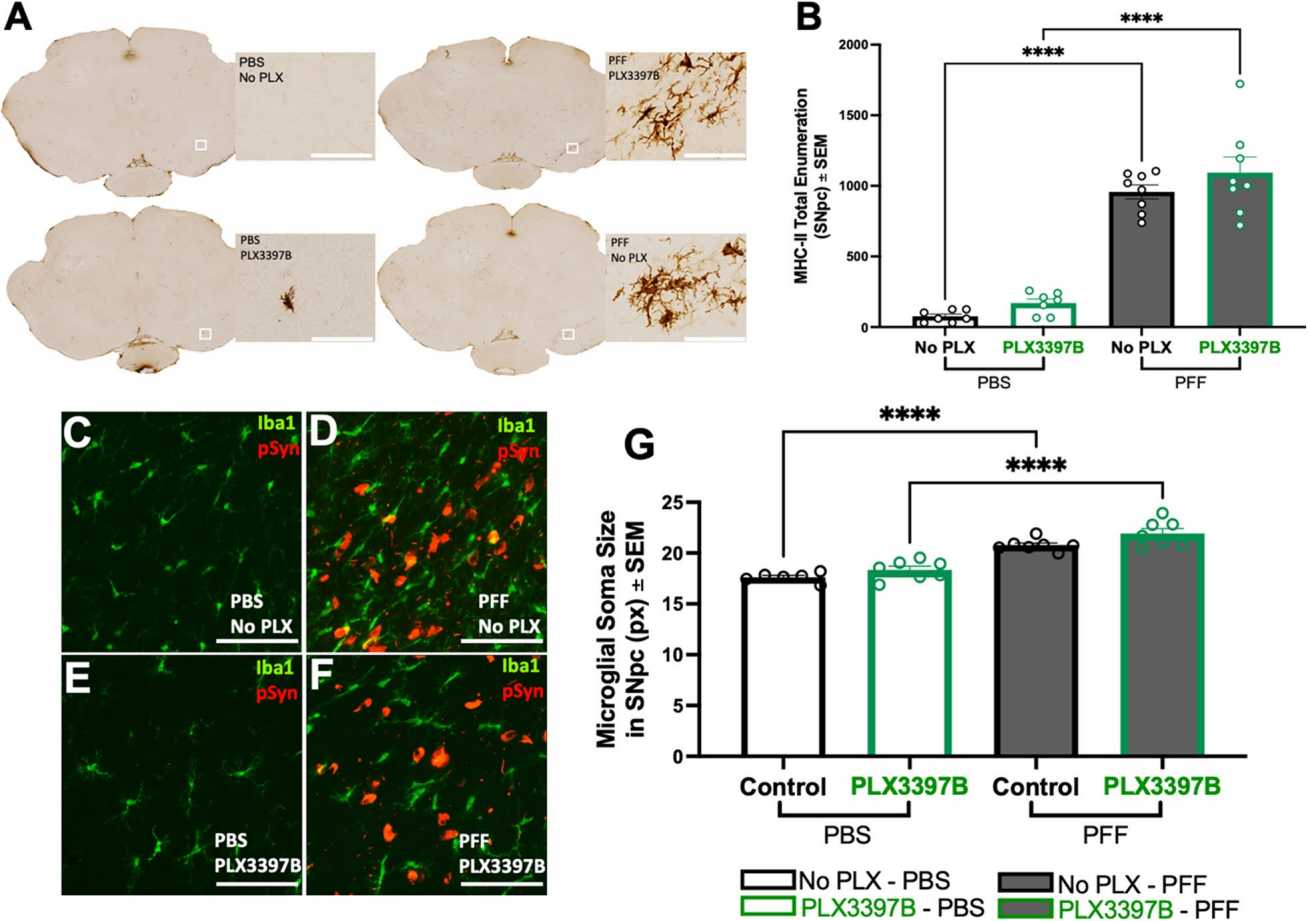
Localized inflammatory response to pSyn inclusions in the SNpc is preserved despite CSF1R inhibition. **A** Major histocompatibility complex II immunoreactive (MHC-IIir) cells in the ipsilateral substantia nigra pars compacta (SNpc) of alpha synuclein preformed fibril (α-syn PFF) or phosphate buffered saline (PBS) injected rats with or without Pexidartinib (PLX3397B) treatment. **B** Quantification of MHC-IIir microglia in the ipsilateral SNpc demonstrates a significant increase in PFF compared to PBS rats at 2 months that is unaffected by PLX3397B treatment. **C**–**F** Ionized calcium-binding adaptor molecule 1 (Iba1, green) and phosphorylated alpha-synuclein at serine 129 (pSyn, red) immunofluorescence in the ipsilateral SNpc 2 months after intrastriatal α-syn PFF or PBS injection, with or without PLX3397B. **G** Quantification of Iba1 immunoreactivity (Iba1ir) microglia soma size demonstrates a significant increase following α-syn PFF injection as compared to PBS that is unaffected by PLX3397B treatment. Values represent the mean ± SEM. Black outline = no PLX3397B; green outline = PLX3397B; ****p < 0.0001. Scale bars in **A** and **C**–**F** 100 µm

pSyn inclusions in the SNpc are associated with an increase in microglial soma size and a localized expression of MHC-II that correlates with α-syn inclusion load [10, 33]. In the present study, we observed numerous MHC-IIir microglia within the SNpc after intrastriatal α-syn PFF injection whereas very few MHC-IIir microglia were observed in PBS control rats (Fig. 4A). Significantly more MHC-IIir microglia were observed in both α-syn PFF control and α-syn PFF PLX3397B SNpc compared to PBS injected rats (p < 0.0001, Fig. 4B). No significant differences were observed in the number of MHC-IIir microglia due to PLX3397B treatment (p > 0.05, Fig. 4B). Similarly, pre and post treatment with Pexidartinib (non-binary) did not lead to a significant difference (p > 0.05)in the number of MHC-IIir microglia within the SNpc (1348 ± 95.75) as compared to rats only receiving Pexidartinib (non-binary) post α-syn PFF injection (1342 ± 84.80) or control fed rats (1167 ± 90.26; Additional file 3: Figure S3B).

Rats with nigral pSyn inclusions exhibited significantly larger microglial soma size in the ipsilateral SNpc compared with microglia in the ipsilateral SNpc of PBS control rats, regardless of PLX3397B treatment (p < 0.0001, Fig. 4C–G). In general, Iba-1 immunoreactive microglia were ~ 15–20% larger in the SNpc of PFF injected rats. No significant differences in microglial soma size were observed within PBS or α-syn PFF treatment groups due to PLX3397B (p > 0.05).

**Fig. 5:**
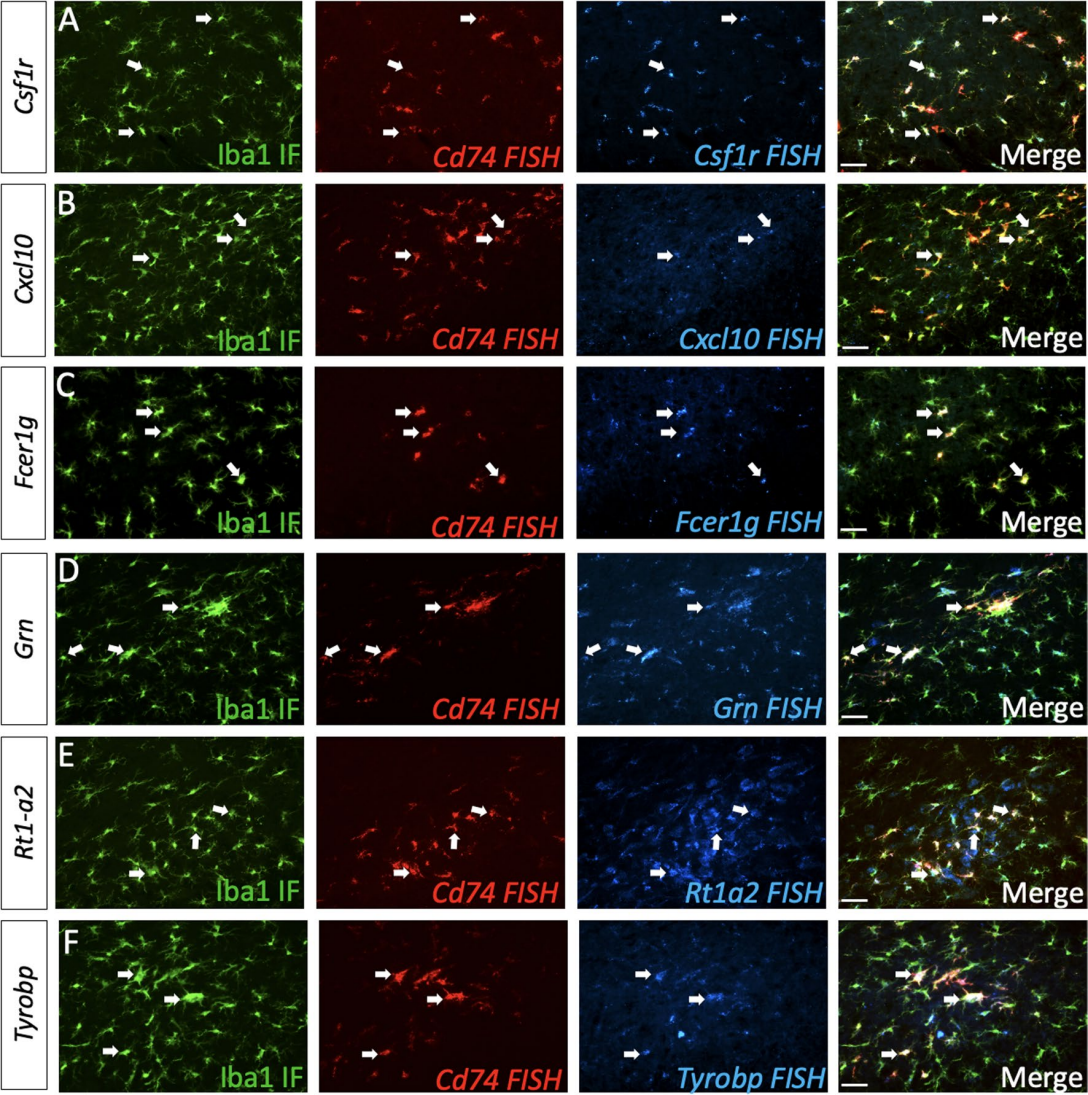
Expression of innate immune genes in microglia proximal to α-syn inclusions is maintained despite CSF1R inhibition. **A** Immunofluorescence (IF) for ionized calcium binding adaptor molecule 1 (Iba1, green) and fluorescent in-situ hybridization (FISH) for *Cd74* (red) and *Csf1r* (blue). **B** IF for Iba1 (green) and FISH for *Cd74* (red) and *Cxcl10* (green). **C** IF for Iba1 (green) and FISH for *Cd74* (red) and *Fcer1g* (green). **D** IF for Iba1 (green) and FISH for *Cd74* (red) and *Grn* (green). **E** IF for Iba1 (green) and FISH for *Cd74* (red) and *Rt1-a2* (green). **F** IF for Iba1 (green) and FISH for *Cd74* (red) and *Tyrobp* (green). All images taken in the ipsilateral SNpc 2 months post alpha-synuclein preformed fibril (α-syn PFF) injection with Pexidartinib (PLX3397B) treatment. Scale bars 50 µm

In previous studies we determined that microglial proximal to nigral pSyn inclusions increase *Cd74* expression along with a suite of innate immune genes from multiple immune pathways including antigen presentation, phagocytosis, T-cell regulation [48]. Using Immunofluorescence (IF) combined with fluorescent in-situ hybridization (FISH), we observed that microglia proximal to pSyn inclusions in PLX3397B treated α-syn PFF injected rats similarly expressed *Cd74, Csf1r, Cxcl10, Fcer1g, Grn, Rt1-a2* and *Tyrobp* (Fig. 5)*.* Collectively, these results suggest that the microglial response to α-syn aggregation is preserved despite CSF1R inhibition and significant depletion of homeostatic microglia.

### Impact of CSF1R inhibition during the nigrostriatal degeneration phase

#### Six months of Pexidartinib (PLX3397B) partially depletes microglia in both α-syn PFF and PBS injected rats

**Fig. 6:**
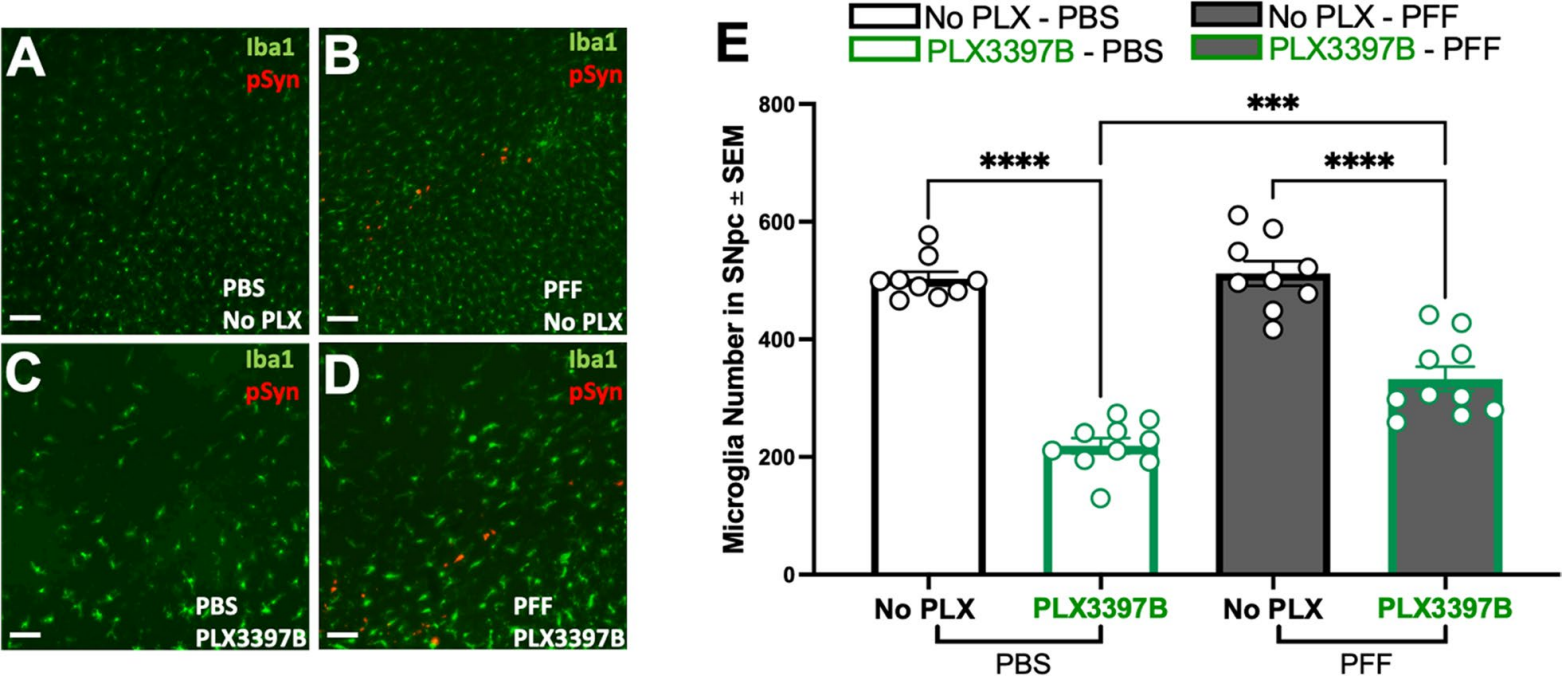
Long term CSF1R inhibition results in significant microglial depletion during nigrostriatal degeneration. **A**–**D** Ionized calcium-binding adaptor molecule 1 (Iba1, green) and phosphorylated alpha-synuclein (pSyn, red) immunofluorescence in the substantia nigra pars compacta (SNpc) 6 months following intrastriatal alpha-synuclein preformed fibril (α-syn PFF) or phosphate buffered saline (PBS) injection, with or without Pexidartinib (PLX3397B). Modest accumulation of pSyn immunoreactive neurons in the ipsilateral SNpc is evident following α-syn PFF injection. **E** Quantitation of Iba1 immunoreactive microglia in the SNpc in all treatment groups. Six months of PLX3397B treatment resulted in significant microglial depletion in both PBS and PFF rats. α-syn PFF PLX3397B rats display significantly more microglia compared to PBS PLX3397B rats. Values represent the mean ± SEM. No PLX3397B = black outline, PLX3397B = green outline. ****p < 0.0001 ***p = 0.0001. Scale bars in **A**–**D** are 100 µm

Α-syn PFF injected rats displayed modest accumulation of pSyn within the SNpc ipsilateral to α-syn PFF injection, however microglia number was not increased due to α-syn PFF injection (p > 0.05, Fig. 6A–E). Similar to the effect of 2 months of PLX3397B treatment, 6 months of PLX3397B led to a significant depletion of Iba-1 immunoreactive microglia in both PBS and α-syn PFF injected rats (Fig. 6E, p < 0.001). PBS PLX3397B rats displayed 56% fewer microglia and α-syn PFF PLX3397B rats displayed 36% fewer microglia compared to control fed rats in their respective surgical treatment groups. Further, PLX3397B α-syn PFF rats possessed significantly more microglia than PLX3397B PBS rats (51% increase, p = 0.001). Our results confirm successful depletion of microglia using PLX3397B over the 6-month interval, although CSF1R inhibition may be somewhat less effective in microglial depletion during nigrostriatal degeneration.

#### CSF1R inhibition does not impact pSyn inclusion triggered degeneration of nigral dopamine neurons

**Fig. 7:**
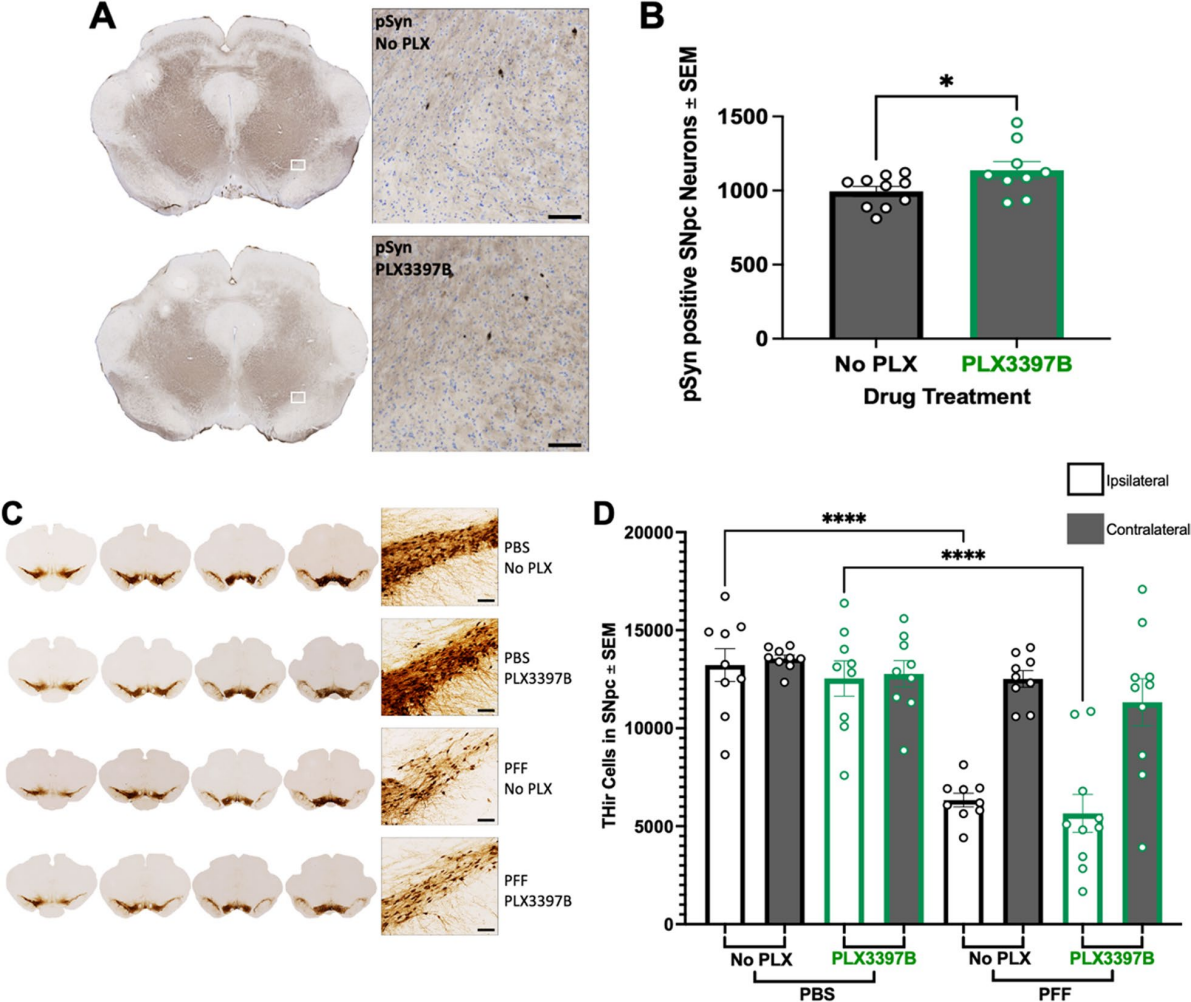
CSF1R inhibition does not impact degeneration of nigrostriatal dopamine neurons following α-synuclein preformed fibril injection. **A** Phosphorylated α-syn (pSyn) inclusions in the ipsilateral substantia nigra pars compacta (SNpc) 6 months post alpha-synuclein preformed fibril (α-syn PFF) injection in both Pexidartinib (PLX3397B) and control fed rats. **B** Quantification of pSyn immunoreactive (pSynir) neurons in the ipsilateral SNpc in rats 6 months after α-syn PFF injection. Significantly fewer pSynir SNpc neurons are observed in α-syn PFF rats fed PLX3397B. **C** Tyrosine hydroxylase immunoreactive (THir) neurons in the SNpc of α-syn PFF and control phosphate buffered saline (PBS) injected rats, with and without 6 months of PLX3397B treatment. **D** Quantification of THir neurons in the SNpc 6 months following surgery. α-syn PFF injection resulted in significant loss of THir SNpc neurons in both PLX3379B and control fed rats. Values represent the mean ± SEM. ****p < 0.0001 *p < 0.05. PLX3397B = green outline, no PLX3397B = black outline. Scale bars in **A** and **C** are 100 µm

Our previous work has demonstrated that few pSyn inclusions remain in the SNpc 6 months following α-syn PFF injection due to the loss of the SNpc neurons that were initially seeded [10, 41]. In general, the number of pSyn immunoreactive (pSynir) SNpc neurons observed at 6 months represents 10–20% of what is observed during the peak 2-month aggregation phase [41]. In the present study we similarly observed an approximate 80% reduction in pSynir neurons in the SNpc at 6 months when compared to 2 months post α-syn PFF injection (p < 0.0001). A modest yet significant increase in pSynir SNpc neurons was observed in α-syn PFF PLX3397B rats compared to α-syn PFF rats fed control chow (p = 0.0470; Fig. 7A, B). We also evaluated the impact of PLX3397B on pSyn accumulation in the striatum, a structure in which pSyn accumulation is abundant at the 6-month time point [41]. No significant differences were observed in pSyn accumulation in the striatum of PFF PLX3397B rats compared to α-syn PFF control chow rats (p > 0.05, Additional file 4: Figure S4). These data suggest that 6 months of PLX3397 treatment results in little to no impact on pSyn accumulation following α-syn PFF injection.

Previous rat α-syn PFF model studies using identical surgical parameters reveal significant loss of ipsilateral SNpc THir neurons 5–6 months post intrastriatal α-syn PFF injection that parallels frank neuronal loss [41]. In the present study, 6 months following surgery, we observed a 52–55% reduction in THir neurons in the ipsilateral SNpc of α-syn PFF rats as compared to the ipsilateral hemisphere of PBS injected rats (p < 0.0001), both with and without PLX3397B treatment (p < 0.0001, Fig. 7C, D). Specifically, the ipsilateral SNpc of α-syn PFF rats fed control chow possessed 6333 ± 349.5 THir neurons whereas the ipsilateral SNpc of PBS rats fed control chow possessed 13,221 ± 838.1 THir neurons. The ipsilateral SNpc of α-syn PFF PLX3397B rats possessed 5658 ± 967.2 THir neurons compared to 12,536 ± 896.8 THir neurons in the ipsilateral SNpc of PBS PLX3397B rats. No significant differences were observed in ipsilateral SNpc THir neurons in α-syn PFF rats due to PLX3397B (p > 0.05). These results suggest that CSF1R inhibition does not impact the loss of THir SNpc neurons during the degeneration phase of the PFF model.

#### Long term CSF1R inhibition results in increased microglia soma size and emergence of MHC-II expression in areas outside the SNpc

**Fig. 8:**
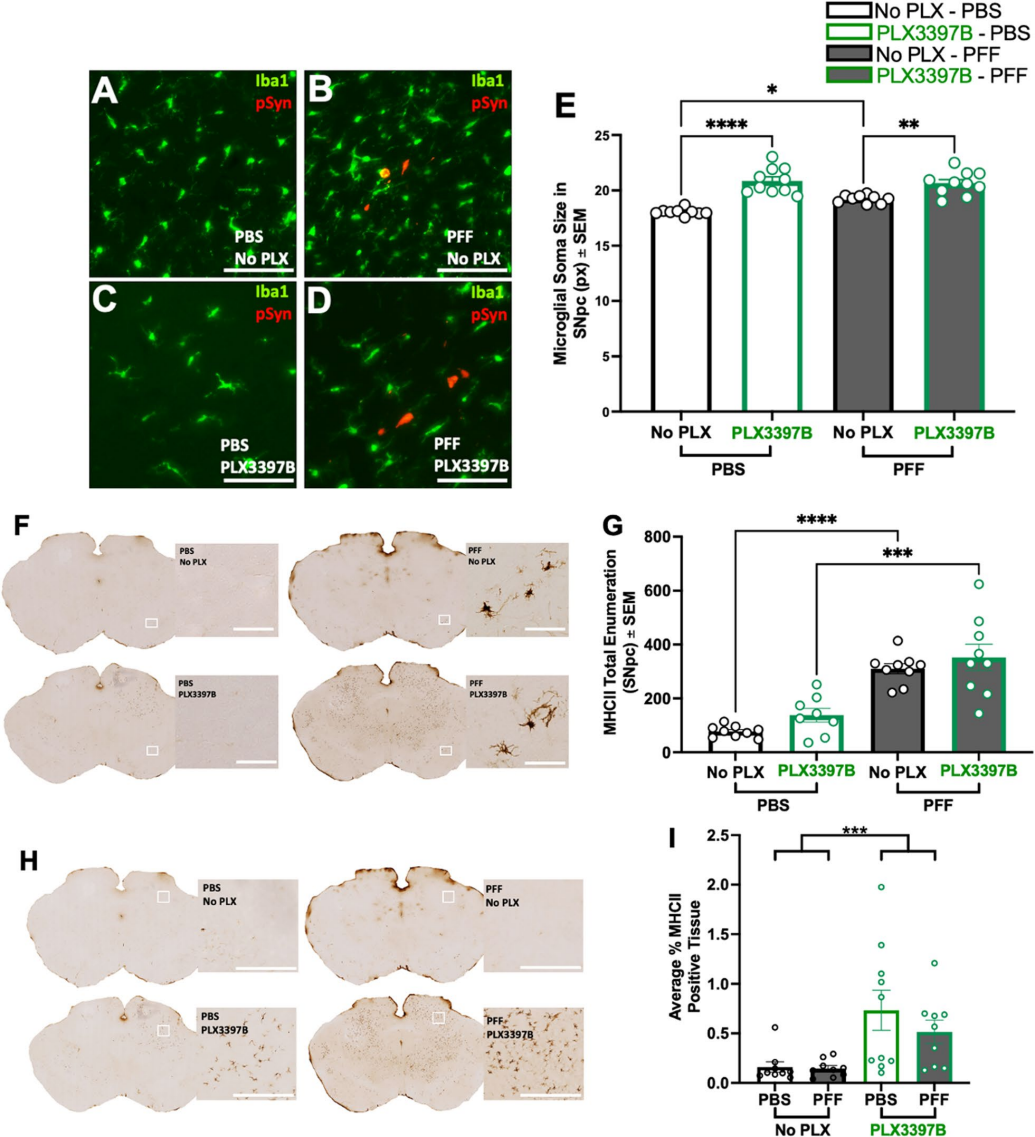
Long-term CSF1R inhibition increases microglial soma size and extranigral major histocompatibility complex II expression. **A**–**D** Ionized calcium-binding adaptor molecule 1 (Iba1, green) and phosphorylated alpha-synuclein (pSyn, red) immunofluorescence in the ipsilateral substantia nigra pars compacta (SNpc) 6 months following intrastriatal alpha-synuclein preformed fibril (α-syn PFF) or phosphate buffered saline (PBS) injection, with or without Pexidartinib (PLX3397B). Modest accumulation of pSyn immunoreactive neurons in the ipsilateral SNpc is evident following α-syn PFF injection. **E** Quantification of microglial soma size demonstrates a significant increase associated with PLX3397B treatment. In rats not fed PLX3397B, α-syn PFF injection is associated with increased microglial soma size. **F** Major histocompatibility complex II immunoreactive (MHC-IIir) microglia in the ipsilateral SNpc of α-syn PFF and PBS injected rats, with and without 6 months of PLX3397B treatment. **G** Quantification of MHC-IIir microglia in the SNpc demonstrates a significant degeneration-associated increase as compared to PBS injected rats at 6 months that is not affected by PLX3397B treatment. **H** MHC-II expression outside of the SNpc in both α-syn PFF and PBS rats after 6 months PLX3397B treatment. **I** Quantification of MHC-IIir expression in the midbrain parenchyma revealed a significant increase associated with long term PLX3397B treatment. Values represent the mean ± SEM. Black outline = no PLX3397B, green outline = PLX3397B. ****p < 0.0001 ***p = 0.001, ** p < 0.01, *p < 0.05. Scale bars in **A**–**D** and **F** are 100 µm. scale bars in **H** are 500 µm

Analysis of the microglial soma size at 6 months revealed that α-syn PFF injected rats possessed significantly larger microglia in the SNpc compared to PBS control rats regardless of PLX3397B treatment (p = 0.0194, Fig. 8A–E). Further, 6 months of CSF1R inhibition led to a significant increase in microglial soma size in both PBS and α-syn PFF injected animals (p < 0.001). We next analyzed the number of MHC-IIir microglia in the SNpc ipsilateral to injection. MHC-IIir microglia peak in abundance in the SNpc 2 months after intrastriatal α-syn PFF injection, in immediate proximity to pSyn inclusions [10]. Although the number of MHC-IIir microglia decrease in abundance over time, MHC-IIir microglia remain elevated compared to controls during the degenerative phase at 6 months [10]. In alignment with these earlier observations, in the present experiment we observed a significant decrease in the number of MHC-IIir microglia in the SNpc of PFF injected rats at 6 months compared to 2 months (p < 0.0001), representing a reduction of approximately 70%. Despite the reduced population of MHC-IIir microglia, we observed a significant increase in MHC-IIir microglia in α-syn PFF rats compared to controls, in both PLX3397B treated (p = 0.0001) and untreated (p < 0.0001) groups (Fig. 8F, G). Specifically, α-syn PFF control chow rats possessed 302% more MHC-IIir microglia than PBS control chow rats, whereas α-syn PFF PLX3397B rats possessed 214% more MHC-IIir microglia than PBS PLX3397B rats. Within surgical treatment groups, no significant differences were observed in the number of MHC-IIir microglia in the SNpc due to PLX3397B treatment (p > 0.05, Fig. 8G). Further, in rats that received PLX3397B chow (both α-syn PFF and PBS) we also noticed MHC-II expression in the mesencephalon outside the nigral region (Fig. 8H). Quantification of MHC-II expression in the extranigral mesencephalon revealed a significant increase associated with long term PLX3397B treatment (p = 0.0006; Fig. 8I). Collectively, these results suggest that despite significant microglial depletion, the localized inflammatory response to nigral degeneration normally observed following α-syn PFF injection is preserved. Further, long term microglial depletion may produce an enhanced proinflammatory phenotype in remaining microglia.

## Discussion

Imaging and histological studies provide support for the presence of ongoing neuroinflammatory processes in PD [30, 31, 34, 37, 46, 50, 58]. Our previous studies have revealed that microglia react to the aggregation and degeneration phases of the rat α-syn PFF model in a consistent, measurable manner [10, 33, 47]. During the peak aggregation phase in the SNpc at 2 months, microglia increase in number, soma size and MHC-II expression. The number of MHC-IIir microglia positively correlates to the number of pSyn immunoreactive SNpc neurons and is markedly decreased during the nigral degeneration phase [10]. During the peak aggregation phase, microglia in the immediate vicinity of SNpc inclusions upregulate *Cd74, Cxcl10, Rt-1a2, Grn, Csf1r, Tyrobp, C3, C1qa, Serping1* and *Fcer1g* [48]. Our present results demonstrate that CSF1R inhibition significantly decreases homeostatic microglia. However, the subpopulation of microglia in the immediate vicinity of pSyn inclusions were resistant to CSF1R inhibition and continued to upregulate MHC-II, *Cd74, Cxcl10, Fcer1g, Grn, Rt1-a2, Tyrobp,* and exhibit increased soma size. Further, CSF1R inhibition did not prevent α-syn aggregation in the SNpc or the striatum or prevent nigral degeneration following intrastriatal PFF injection.

In the present experiment we initially expected that CSF1R inhibition would decrease all microglia, including both homeostatic and pSyn inclusion responsive microglia. The maintenance of the pSyn inclusion responsive microglial subpopulation, despite CSF1R inhibition, suggests that this subpopulation is not dependent on CSF1R activation for survival. Interestingly, a similar phenomenon has previously been described in studies examining the effect of CSF1R inhibition during retinal development. Specifically, homeostatic microglia were revealed to be dependent on CSF1R activation, however microglia responding to neuronal apoptosis did not depend on CSF1R activation for survival [1]. Additional studies using the CSF1R inhibition approaches have reported a similar maintenance or an increased inflammatory response, despite depletion of the general microglial population [4, 12, 13] along with increases in adaptive immune cells within the brain [59]. Additional studies will be required to understand the mechanism whereby pSyn inclusion responsive microglia become CSF1R activation independent. Insights into this mechanism may provide clues for future therapeutic intervention.

The ability to attenuate inflammatory processes through CSF1R inhibition has yielded mixed results in both AD (Tau; [4] and PD (MPTP [38]) animal models. In some studies, CSF1R inhibition has led to the exacerbation of neurodegeneration [21, 25, 60] whereas in others neuroprotection is observed [38, 40]. Previous studies using CSF1R inhibitors in mice employed dosing strategies that resulted in near complete microglial depletion (~ 90%) [13, 16, 40]. However, microglia play many roles in maintaining healthy homeostasis in the brain [23, 32, 49] and thus complete microglia depletion may not be a safe therapeutic strategy. Therefore, in the present study we employed a PLX3397B dosing strategy that elicited partial (~ 40%) microglial depletion in the SNpc in the ⍺-syn PFF model.

Findings support a bidirectional relationship between microglia and ⍺-syn aggregation. ⍺-syn aggregation in the PFF model is associated with increased microglial soma size and MHC-II expression [10]. Microglia can degrade neuron-derived ⍺-synuclein and inhibition of microglial autophagy can lead to increased ⍺-synuclein aggregation [6]. Amplification of the NLRP3 inflammasome increases monomeric ⍺-syn levels and accelerates the formation of PFF-triggered aggregates [61]. In our present study, despite significant homeostatic microglial depletion, the MHC-II immunoreactive microglial subpopulation associated with ⍺-syn aggregation was maintained. Given that the magnitude of ⍺-syn aggregation and nigrostriatal degeneration was unchanged by CSF1R inhibition, a more comprehensive understanding of the role of the responding microglia is warranted. In addition, studies using ⍺-syn overexpression models, which display a different neuroinflammatory profile and magnitude of MHC-II expression [9, 28], would add further insight into the role of microglia in neurodegeneration*.*

The approach of microglia repopulation as a therapeutic strategy in order to “reset” microglia has been recently proposed with the goal of exchanging dysfunctional with functional microglia. However, the results from repopulation studies vary [2, 16] and suggest that repopulation comes from the remaining microglia. Our data suggest a microglia repopulation strategy would not be beneficial, and that the inflammatory response to pSyn inclusions and nigrostriatal degeneration would be maintained.

Our study is unique in that the CSF1R inhibition was sustained for a period of 6 months, whereas most previous CSF1R inhibition studies use much shorter depletion intervals (7–28 days [2, 12, 14, 16, 60]. We observed evidence of a more pronounced inflammatory state in our 6-month CSF1R inhibition study compared to our 2-month CSF1R inhibition study. Specifically, after 6 months of CSF1R inhibition, microglia soma size was increased, even within control PBS injected rats. Further, after 6 months of CSF1R inhibition we observed MHC-IIir cells in multiple brain regions, and also in control rats. Normally, except for border associated macrophages [54, 55], MHC-IIir cells are not often observed in uninjured brain regions in control rats. The increased MHC-II expression we observe with long term CSF1R inhibition may be attributable to microglia or to infiltrating monocytes, border associated macrophages or perivascular macrophages [3, 15, 18, 53]. It is unclear whether this increased MHC-II expression in these extranigral regions exerted any neurotoxic effects. Future investigation is required to ascertain the identity of the cells that respond to CSF1R inhibition with upregulated MHC-II expression, as well as whether any detrimental consequences result from this increase in MHC-II.

## Conclusions

Inflammatory microglia may contribute to PD progression and microglial based inflammation has been under investigation in order to identify therapeutic targets. One limitation of the present study is that the response of other cell types (peripheral macrophages, astrocytes, adaptive immune cells) to CSF1R inhibition was not examined. Previous studies have indicated that near complete microglial depletion can impact astrocytes and adaptive immune cells [4]. Another limitation of the present study is that the magnitude of microglial depletion was not that which has been previously achieved in mouse studies (~ 90%) [13, 16, 40]. It is possible that near complete levels of microglial depletion may have yielded different outcomes. Despite these limitations, the present study suggests that CSF1R inhibition may not be an effective, disease-modifying approach for PD and may instead induce a heightened proinflammatory state in remaining microglia.

### Supplementary Information

**Additional file 1: Figure S1.** Chow consumption, rat weight change and liver weights after 2 months of PLX3397B treatment. **A:** Food consumption each week in all four rat treatment groups over 2 months post-surgery. **B:** Average weight change in all four rat treatment groups. **C:** Liver weights at time of euthanasia in all 4 rat treatment groups. Values represent the mean ± SEM. Black outline = no PLX3397B, green outline = PLX3397B. PFF = alpha-synuclein preformed fibrils, PBS = phosphate buffered saline, PLX = PLX3397B.

**Additional file 2: Figure S2.** Chow consumption, rat weight change and liver weights after 6 months of PLX3397B treatment. **A:** Food consumption each week in all four rat treatment groups over 6 months post-surgery. **B:** Average weight change in all four rat treatment groups. **C:** Liver weights at time of euthanasia in all 4 rat treatment groups. Values represent the mean ± SEM. Black outline = no PLX3397B, green outline = PLX3397B. PFF = alpha-synuclein preformed fibrils, PBS = phosphate buffered saline, PLX = PLX3397B.

**Additional file 3: Figure S3.** pSyn aggregation and localized inflammatory response to pSyn inclusions in the SNpc is preserved despite Pexidartinib pretreatment. **A:** Quantification of phosphorylated alpha-synuclein (α-syn) immunoreactive (pSynir) neurons in the ipsilateral substantia nigra pars compacta (SNpc) 2 months post α-syn preformed fibril (α-syn PFF) injection in rats fed control chow, Pexidartinib (non-binary) chow pre and post surgery, and Pexidartinib chow post surgery only. Pexidartinib (non-binary) treatment, either pre and post surgery or post surgery only, did not impact on the number of pSynir neurons within the SNpc. **B:** Quantification of major histocompatibility complex II immunoreactive (MHC-IIir) microglia in the ipsilateral SNpc 2 months after α-syn PFF injection in control. Pexidartinib (non-binary) treatment, either pre and post surgery or post surgery only, did not impact the number of MHC-IIir microglia within the SNpc.

**Additional file 4: Figure S4.** CSF1R inhibition for 6 months does not impact accumulation of phosphorylated alpha-synuclein in the striatum. Quantification of phosphorylated alpha-synuclein (pSyn) accumulation in the striatum 6 months following intrastriatal alpha-synuclein preformed fibril (α-syn PFF) in Pexidartinib (PLX3397B) rats compared to rats that were fed control chow. No significant difference was seen in striatal pSyn load between treatment groups.

**Additional file 5: Table S1.**  Detailed FISH probe information.

## Data Availability

The datasets supporting the conclusions of this article are available from the corresponding author upon reasonable request.

## Abbreviations

α-syn: Alpha-synuclein
AAALAC: Association for Assessment and Accreditation of Laboratory Animal Care
ANOVA: Analysis of variance
DA: Dopamine
DAPI: 4ʹ,6-Diamidino-2-phenylindole, dihydrochloride
CSF: Cerebral Spinal fluid
CSF1R: Colony stimulating factor 1 receptor
HLA-DR: Human leukocyte antigen-antigen D related
IACUC: Institutional Animal Care and Use Committee
Iba1: Ionized calcium binding adaptor 1
IF: Immunofluorescence
IHC: Immunohistochemistry
ir: Immunoreactive
MHC-II: Major histocompatibility complex II
mPFFs: Mouse preformed fibrils
NGS: Normal goat serum
PB: Phosphate Buffer
PBS: Phosphate buffered saline
PD: Parkinson’s disease
PFA: Paraformaldehyde
PFFs: Preformed fibrils
PLX3397B: Pexidartinib binary
pSyn: Phosphorylated alpha-synuclein at SER 129
SN: Substantia nigra
SNpc: Substantia nigra pars compacta
STR: Striatum
TBS: Tris buffered saline
TH: Tyrosine hydroxylase
Tx-100: Triton X-100
