# Supplementary figures and images for: Alpha-synuclein inclusion responsive microglia are resistant to CSF1R inhibition
Source: J Neuroinflammation. 2024 Apr 25;21:108. doi: 10.1186/s12974-024-03108-5 (PMC11045433; doi:10.1186/s12974-024-03108-5)

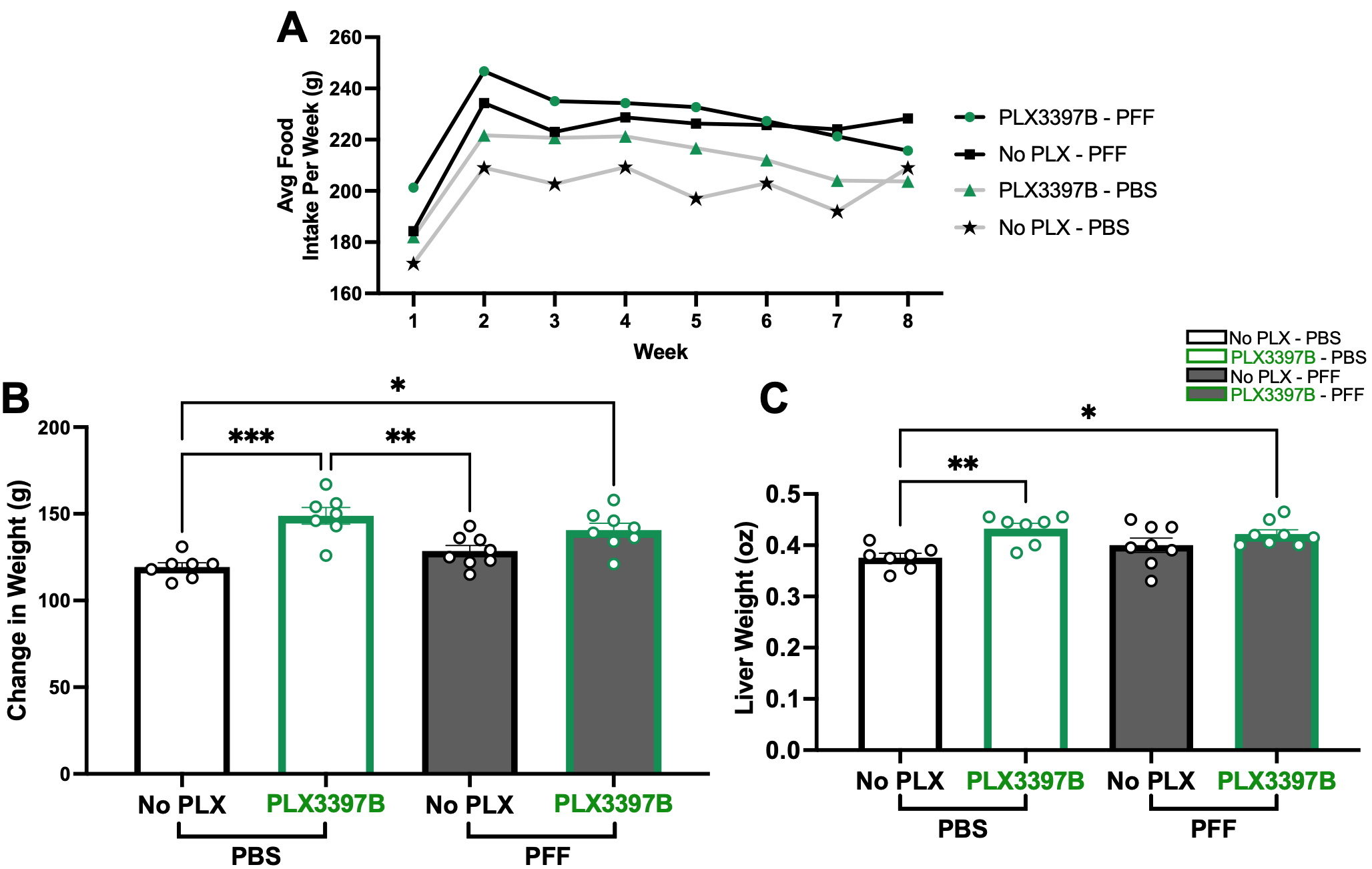

Supplement: Supplementary file 1 — Additional file 1: Figure S1. Chow consumption, rat weight change and liver weights after 2 months of PLX3397B treatment. A: Food consumption each week in all four rat treatment groups over 2 months post-surgery. B: Average weight change in all four rat treatment groups. C: Liver weights at time of euthanasia in all 4 rat treatment groups. Values represent the mean ± SEM. Black outline = no PLX3397B, green outline = PLX3397B. PFF = alpha-synuclein preformed fibrils, PBS = phosphate buffered saline, PLX = PLX3397B. [file 12974_2024_3108_MOESM1_ESM.tiff]

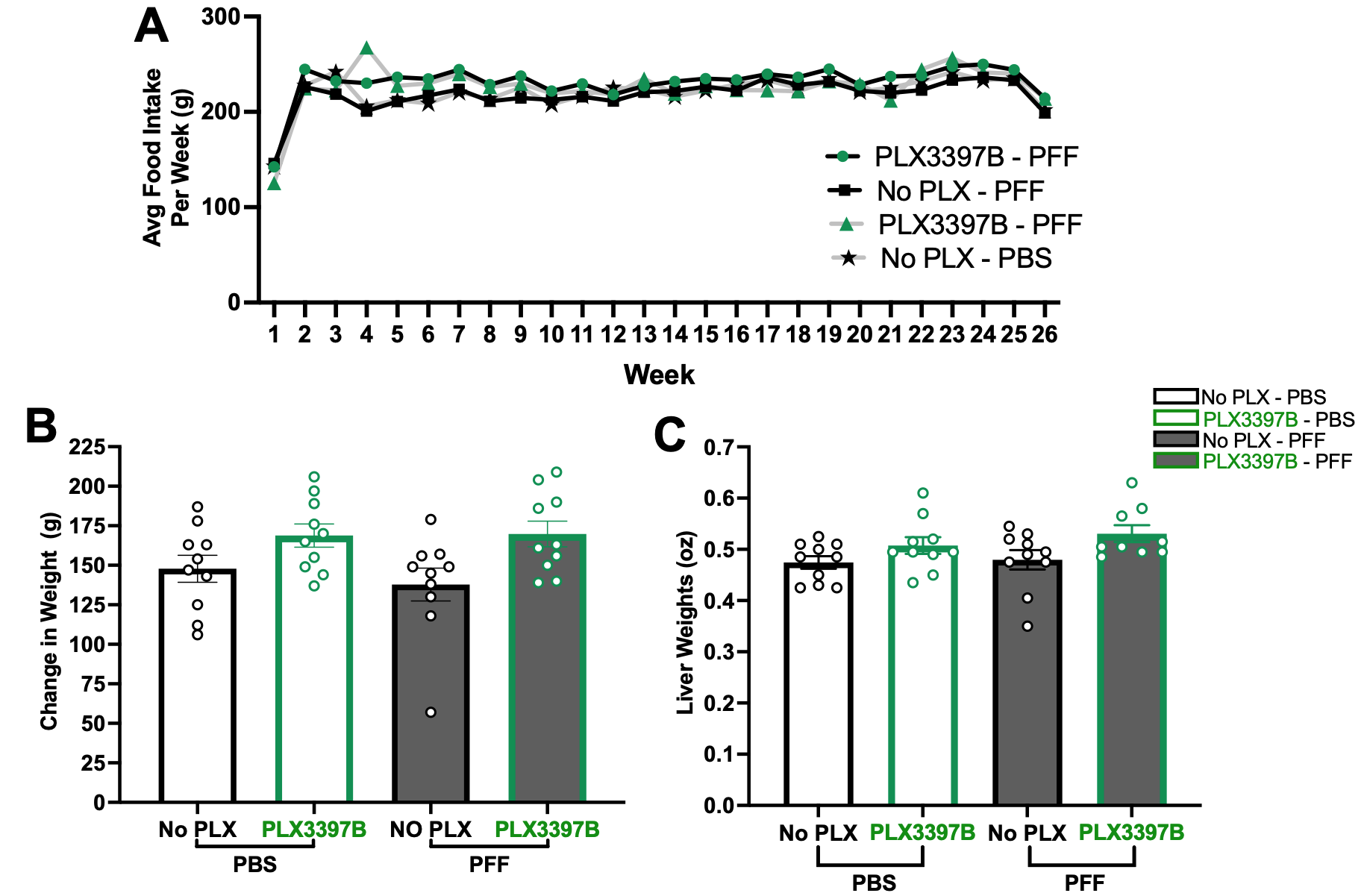

Supplement: Supplementary file 2 — Additional file 2: Figure S2. Chow consumption, rat weight change and liver weights after 6 months of PLX3397B treatment. A: Food consumption each week in all four rat treatment groups over 6 months post-surgery. B: Average weight change in all four rat treatment groups. C: Liver weights at time of euthanasia in all 4 rat treatment groups. Values represent the mean ± SEM. Black outline = no PLX3397B, green outline = PLX3397B. PFF = alpha-synuclein preformed fibrils, PBS = phosphate buffered saline, PLX = PLX3397B. [file 12974_2024_3108_MOESM2_ESM.tiff]

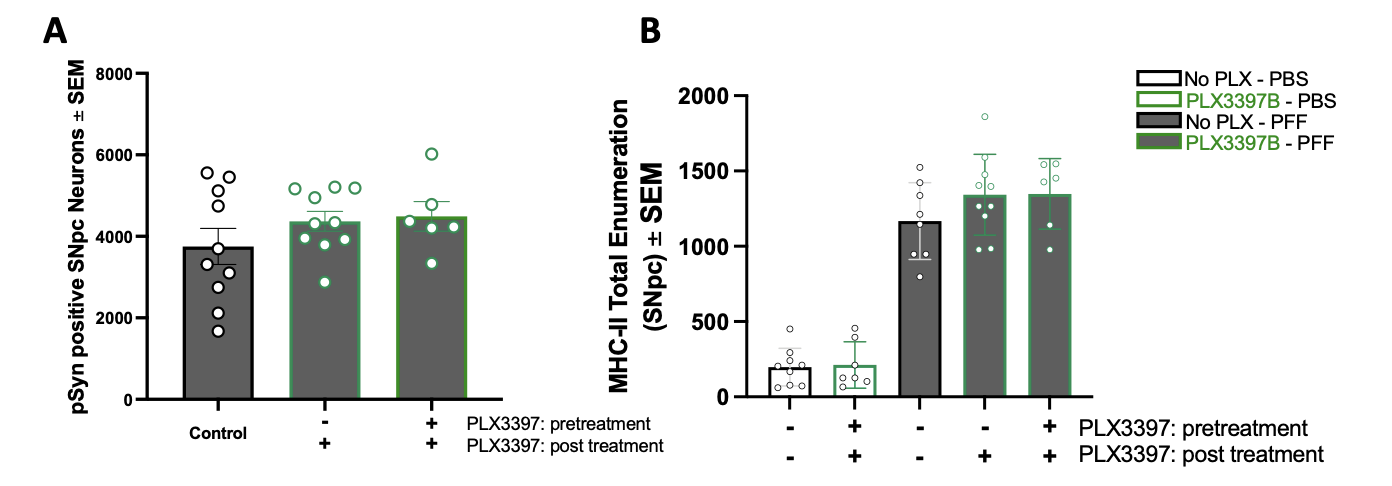

Supplement: Supplementary file 3 — Additional file 3: Figure S3. pSyn aggregation and localized inflammatory response to pSyn inclusions in the SNpc is preserved despite Pexidartinib pretreatment. A: Quantification of phosphorylated alpha-synuclein (α-syn) immunoreactive (pSynir) neurons in the ipsilateral substantia nigra pars compacta (SNpc) 2 months post α-syn preformed fibril (α-syn PFF) injection in rats fed control chow, Pexidartinib (non-binary) chow pre and post surgery, and Pexidartinib chow post surgery only. Pexidartinib (non-binary) treatment, either pre and post surgery or post surgery only, did not impact on the number of pSynir neurons within the SNpc. B: Quantification of major histocompatibility complex II immunoreactive (MHC-IIir) microglia in the ipsilateral SNpc 2 months after α-syn PFF injection in control. Pexidartinib (non-binary) treatment, either pre and post surgery or post surgery only, did not impact the number of MHC-IIir microglia within the SNpc. [file 12974_2024_3108_MOESM3_ESM.tiff]

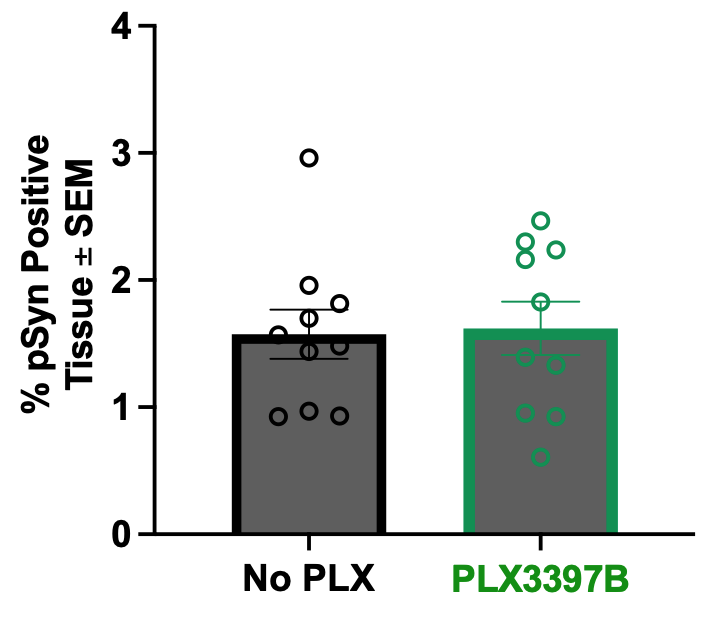

Supplement: Supplementary file 4 — Additional file 4: Figure S4. CSF1R inhibition for 6 months does not impact accumulation of phosphorylated alpha-synuclein in the striatum. Quantification of phosphorylated alpha-synuclein (pSyn) accumulation in the striatum 6 months following intrastriatal alpha-synuclein preformed fibril (α-syn PFF) in Pexidartinib (PLX3397B) rats compared to rats that were fed control chow. No significant difference was seen in striatal pSyn load between treatment groups. [file 12974_2024_3108_MOESM4_ESM.tiff]

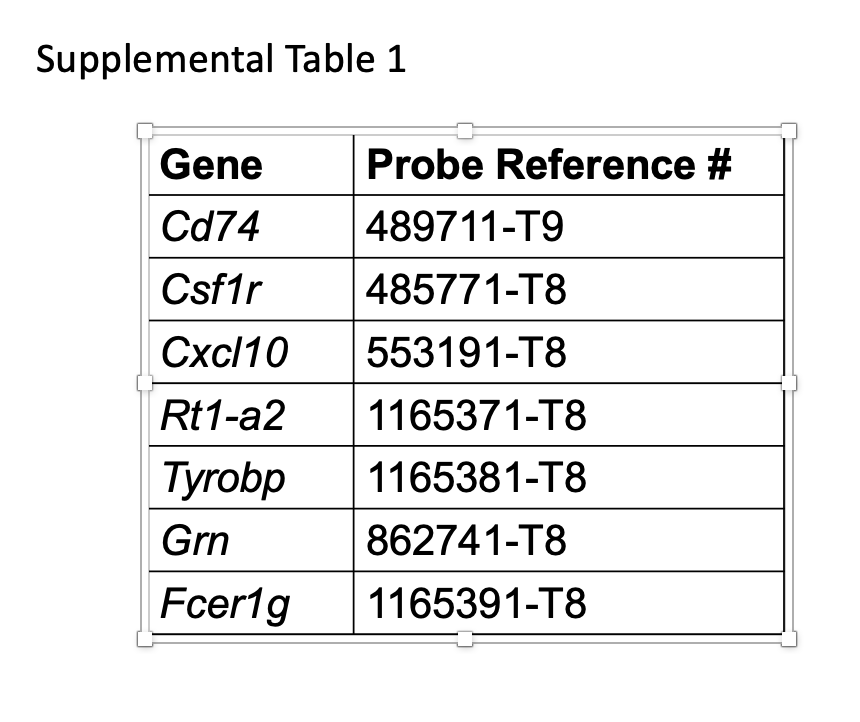

Supplement: Supplementary file 5 — Additional file 5: Table S1. Detailed FISH probe information. [file 12974_2024_3108_MOESM5_ESM.tiff]
